# A large ovarian steroid cell tumor‐not otherwise specified with a unique combination of benign and malignant features as a challenging cause of oligomenorrhea and hirsutism in a 21‐year‐old Syrian female: a case report

**DOI:** 10.1186/s12905-021-01244-1

**Published:** 2021-03-04

**Authors:** Sawsan Ismail, Munawar Hraib, Rana Issa, Thanaa Alassi, Zuheir Alshehabi

**Affiliations:** 1 Department of Pathology, Cancer Research Center, Faculty of Medicine, Tishreen University, Lattakia, Syria; 2 Faculty of Medicine, Tishreen University, Lattakia, Syria; 3 Department of Pathology, Faculty of Medicine, Tishreen University, Lattakia, Syria; 4 Department of Gynecology, Alsaydeh Surgical Hospital, Lattakia, Syria

**Keywords:** Steroid cell tumors, Ovarian neoplasms, Oligomenorrhea, Histopathology

## Abstract

**Background:**

Ovarian steroid cell tumors represent a rare category of sex cord-stromal tumors that constitute less than 0.1% of all ovarian tumors. These neoplasms are classified into three main subtypes according to the cell of origin: Leidyg cell tumors, stromal luteomas, and steroid cell tumors not otherwise specified (SCTs-NOS). The latter subtype is defined as a neoplasm of an uncertain lineage that mostly affects middle-aged women, whereas it’s rare in younger ages.

**Case presentation:**

We report a case of a 21-year-old virgin female who presented to our hospital with complaints of mild abdominal pain, hirsutism, and oligomenorrhea for more than a year. Before her current admission, the patient had attended an external gynecologic clinic where she had been prescribed oral contraceptives to regulate her periods. Nevertheless, on presentation to our institution, physical examination revealed abdominal tenderness with a palpable pelvic mass and mild hirsutism in the thigh. Ultrasonography demonstrated a large left ovarian mass measuring 154 × 104 mm, and compressing the uterus. Therefore, a unilateral salpingo-oophorectomy was performed, and interestingly, pathologic examination of the large aforementioned mass alongside with immunohistochemical correlation revealed the diagnosis of a large ovarian steroid cell tumor-not otherwise specified with a unique combination of benign and malignant features.

**Conclusions:**

Although ovarian steroid cell tumors represent a rare category, they must be considered in the differential diagnosis for mild virilization symptoms in young females due to the importance of early diagnosis and management. In this manuscript, we aimed to present the first case report from Syria that highlights the crucial role of detailed morphological examination for challenging cases despite the difficulties in differential diagnosis, and the absence of ancillary techniques. Furthermore, we managed to discuss a brief review of diagnostic methods, histological characteristics, and treatment recommendations.

## Introduction

Ovarian steroid cell tumors are defined as a rare category of sex cord-stromal tumors that constitute less than 0.1% of all ovarian tumors [1]. These neoplasms were historically defined as “virilizing lipid or lipoid cell tumors” in the Atlas of Ovarian Tumors from 1943 [2]. Later, the term steroid cell tumor was suggested by Scully in 1977 due to the high similarities of neoplastic cells to steroid hormone-producing cells, as well as the lack of lipid secretions in approximately 25% of cases [3].

Steroid cell tumors are classified into three main subtypes according to the cell of origin: Leidyg cell tumors, stromal luteomas, and steroid cell tumors not otherwise specified (SCT-NOS) [1, 4]. Hayes and Scully defined the latter subtype as a neoplasm of an uncertain lineage that constitutes approximately 56% of all SCTs, and mostly affects middle-aged women [1]. Herein, we present—to our knowledge—the first Syrian case of a large ovarian steroid cell tumor in a young female with a challenging history.

## Case presentation

A 21-year-old virgin female presented to our hospital with complaints of mild abdominal pain, hirsutism, and oligomenorrhea. Her medical history was of interest. The patient had menarche at the age of 13. However, 1 year before admission to our institution, she experienced hirsutism and oligomenorrhea with menstrual cycles of more than 60 days apart. Therefore she visited an external gynecologic clinic where she was prescribed oral contraceptives to regulate her periods. The patient claimed to observe a mild regression in the symptoms with no further examinations. However, details and data supporting her previous admission were not available. The patient’s mother had a hysterectomy several years ago due to the diagnosis of multiple leiomyomas. Other than that, family and medical history were unremarkable.

**Fig. 1:**
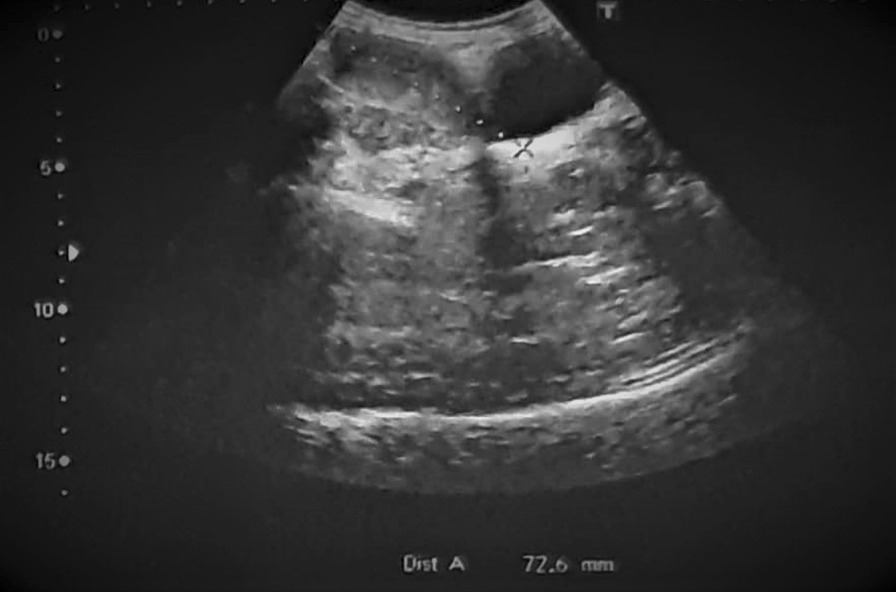
Pelvic ultrasonography demonstrating a large ovarian mass measuring 154 × 104 mm

**Fig. 2:**
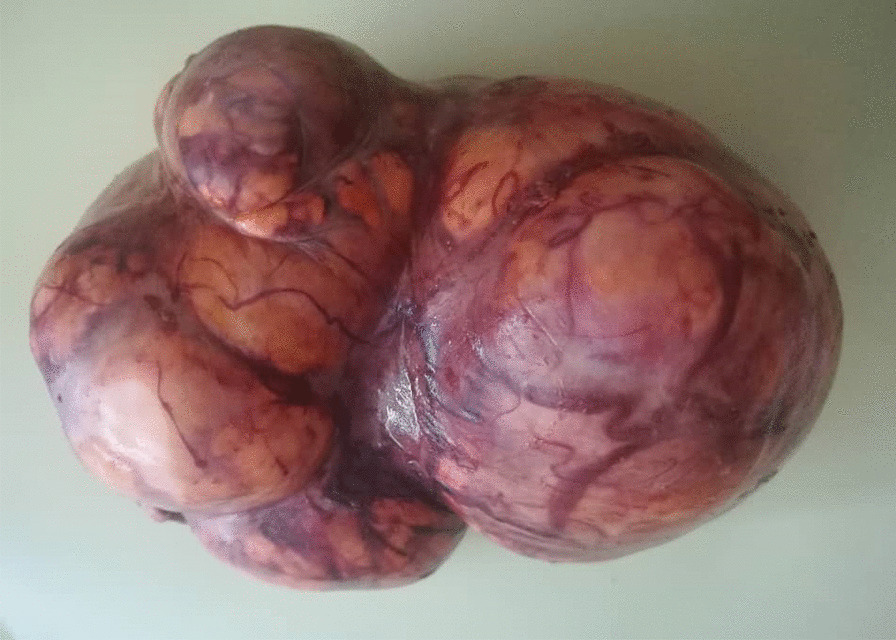
A macroscopic image revealing a large, well-circumscribed lobulated solid yellow-brownish mass, measuring approximately 15 × 9 cm

**Fig. 3:**
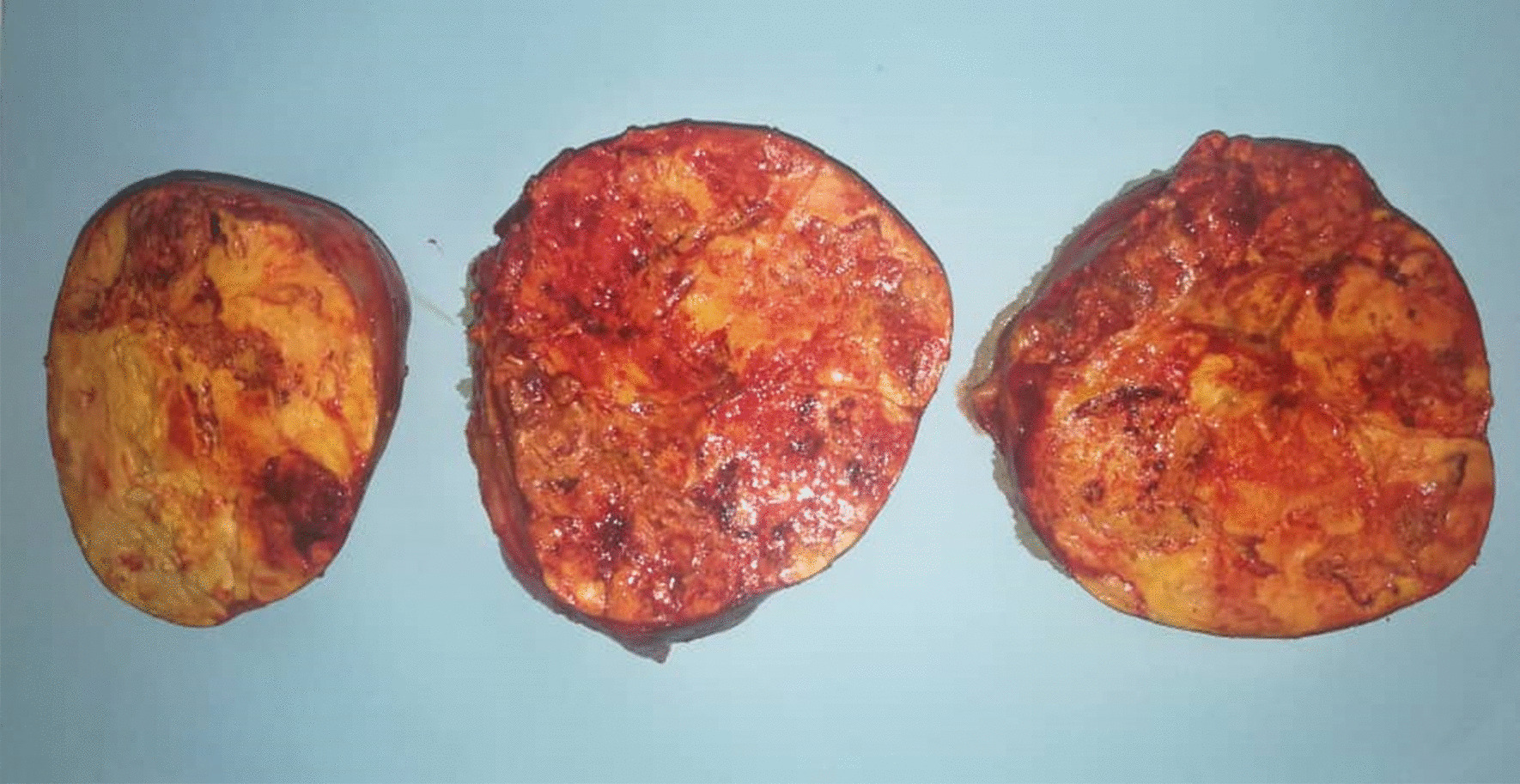
A macroscopic image of cut sections revealing bright orange-yellowish nodules with scattered foci of necrosis and hemorrhage and a rich-vasculature capsule

On presentation to our institution, physical examination revealed abdominal tenderness with a palpable pelvic mass and mild hirsutism in the thigh. Her body mass index (BMI) was 19.1 kg/m² (height: 150 cm, weight: 43 kg). Interestingly, ultrasonography demonstrated a large left ovarian mass measuring 154 × 104 mm, and compressing the uterus (Fig. 1). No pelvic enlarged lymph nodes or Douglas pouch effusion were observed, and computed tomography (CT) scan revealed no other lesions. Due to the large size of the ovarian mass and the risk of compressing the uterus and the adjacent organs, a unilateral salpingo-oophorectomy was performed. Macroscopic examination revealed a large, well-circumscribed lobulated solid yellow-brownish mass, measuring approximately 15 × 9 cm (Fig. 2). Cut sections demonstrated bright orange-yellowish nodules with scattered foci of necrosis and hemorrhage and a rich-vasculature capsule (Fig. 3).

**Fig. 4:**
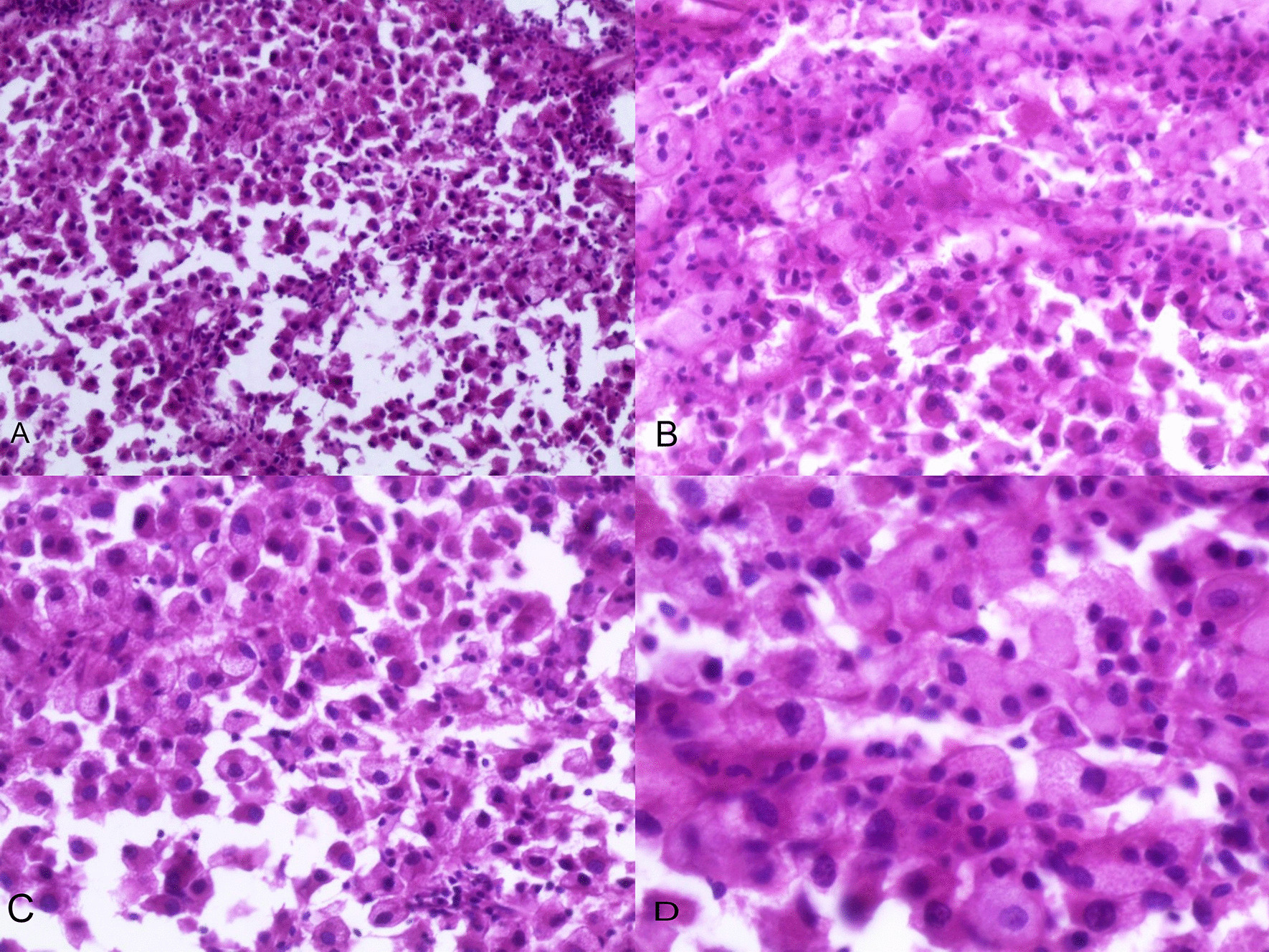
Morphologic features demonstrating diffuse and nodular proliferation of medium-sized to large polygonal neoplastic cells with pale to granular eosinophilic cytoplasm, small round nuclei. (Hematoxylin and Eosin (H&E) stain **a** original magnification × 40, **b** original magnification × 200, **c** original magnification × 200, **d** original magnification × 400)

**Fig. 5:**
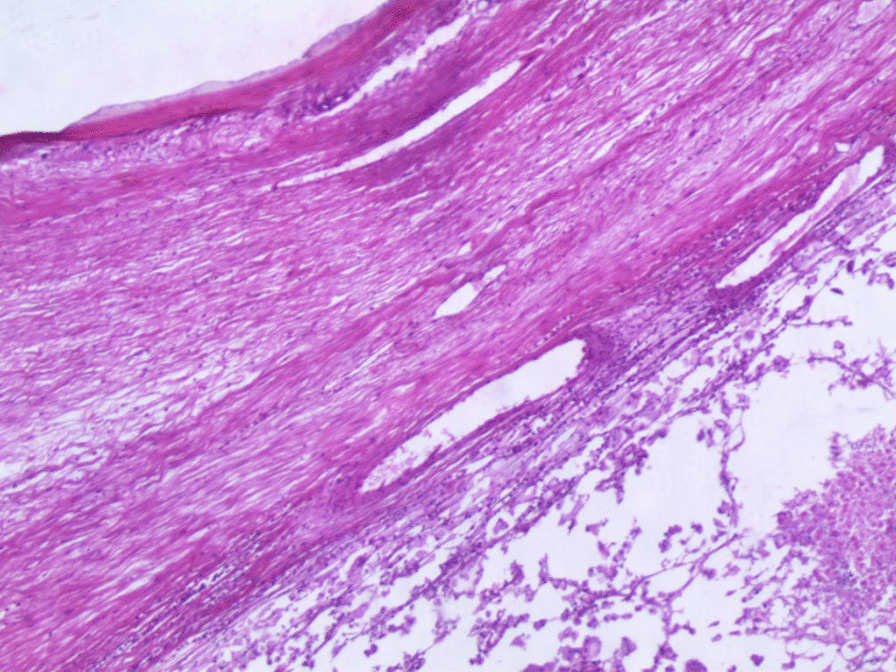
No evidence of capsular invasion. ( H&E stain: original magnification × 40)

**Fig. 6:**
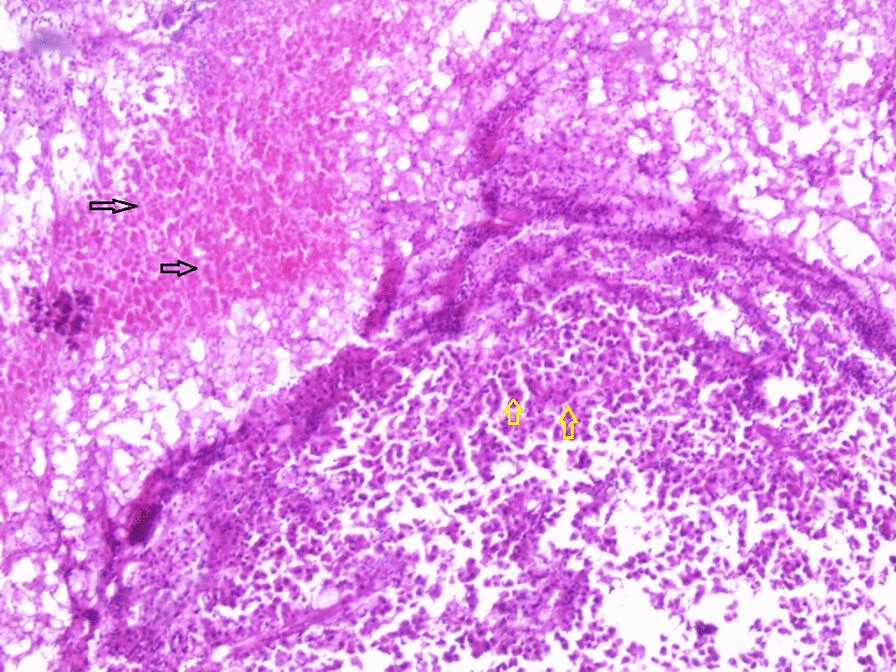
A microscopic image demonstrating scattered foci of necrosis and hemorrhage (black arrows) and the neoplastic cells (yellow arrows). ( H&E stain: original magnification × 40)

**Fig. 7:**
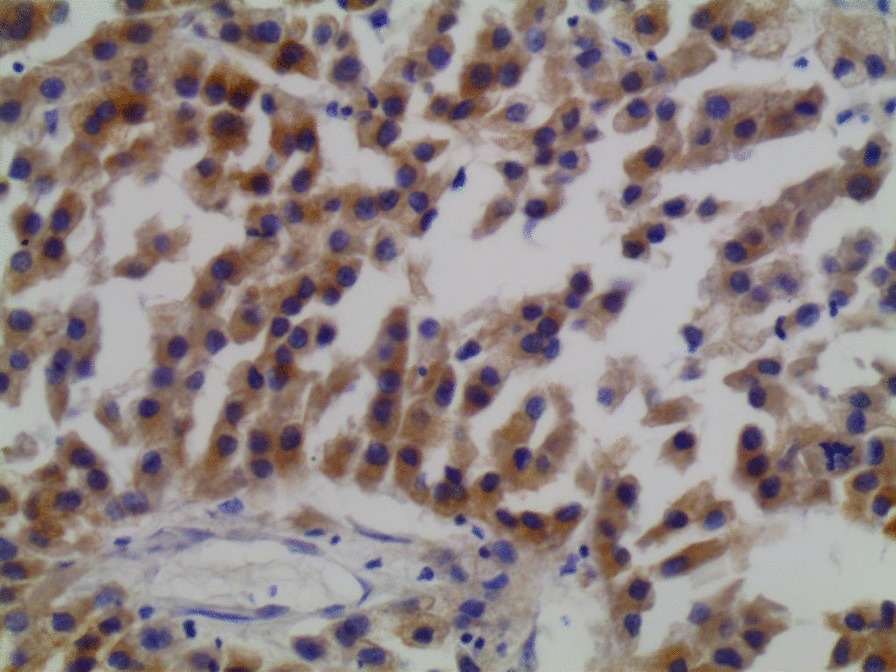
Immunohistochemistry of the neoplasm: high positivity for Inhibin

**Fig. 8:**
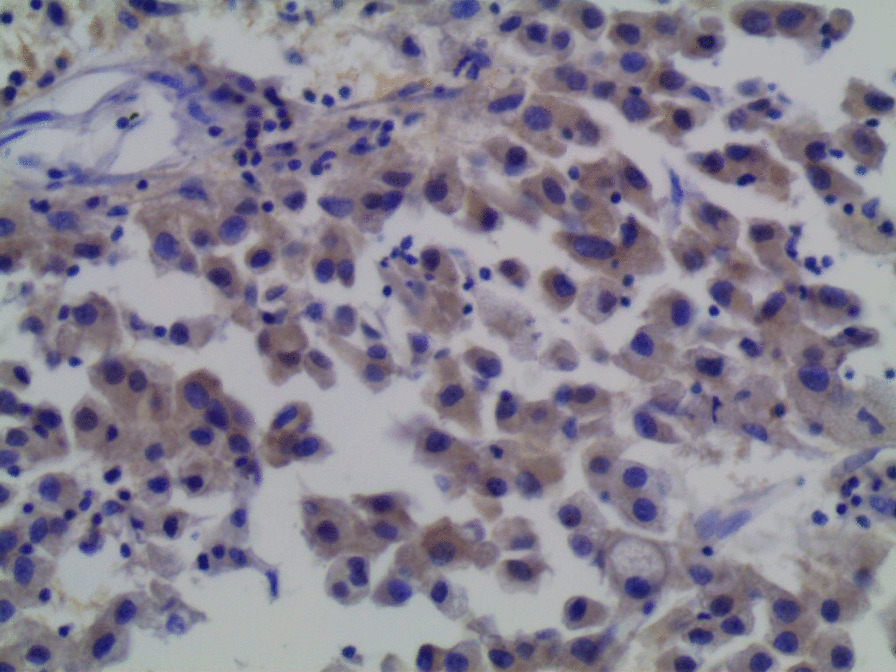
Immunohistochemistry of the neoplasm: high positivity for Calretinin

**Fig. 9:**
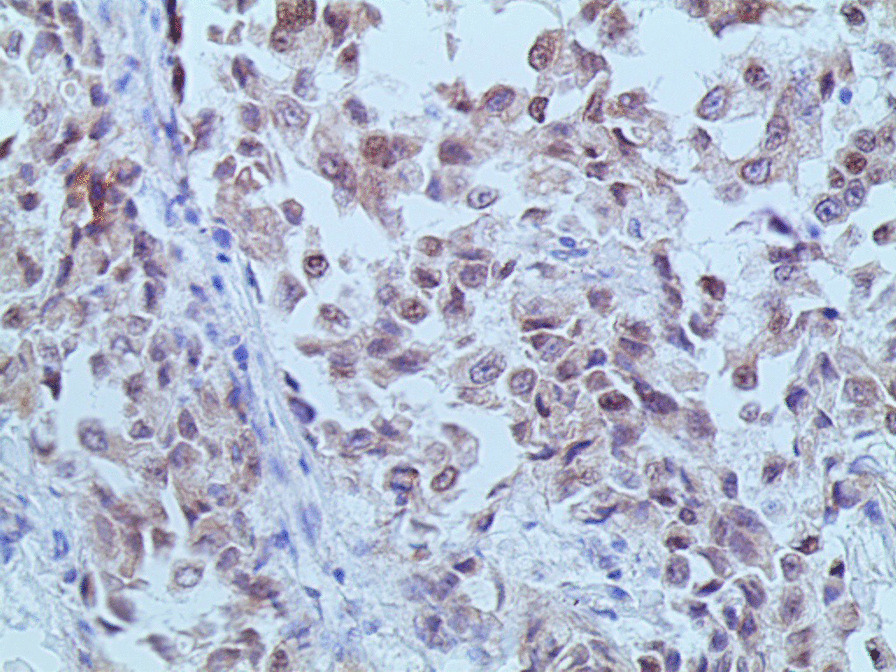
Immunohistochemistry of the neoplasm: positivity for ER

**Fig. 10:**
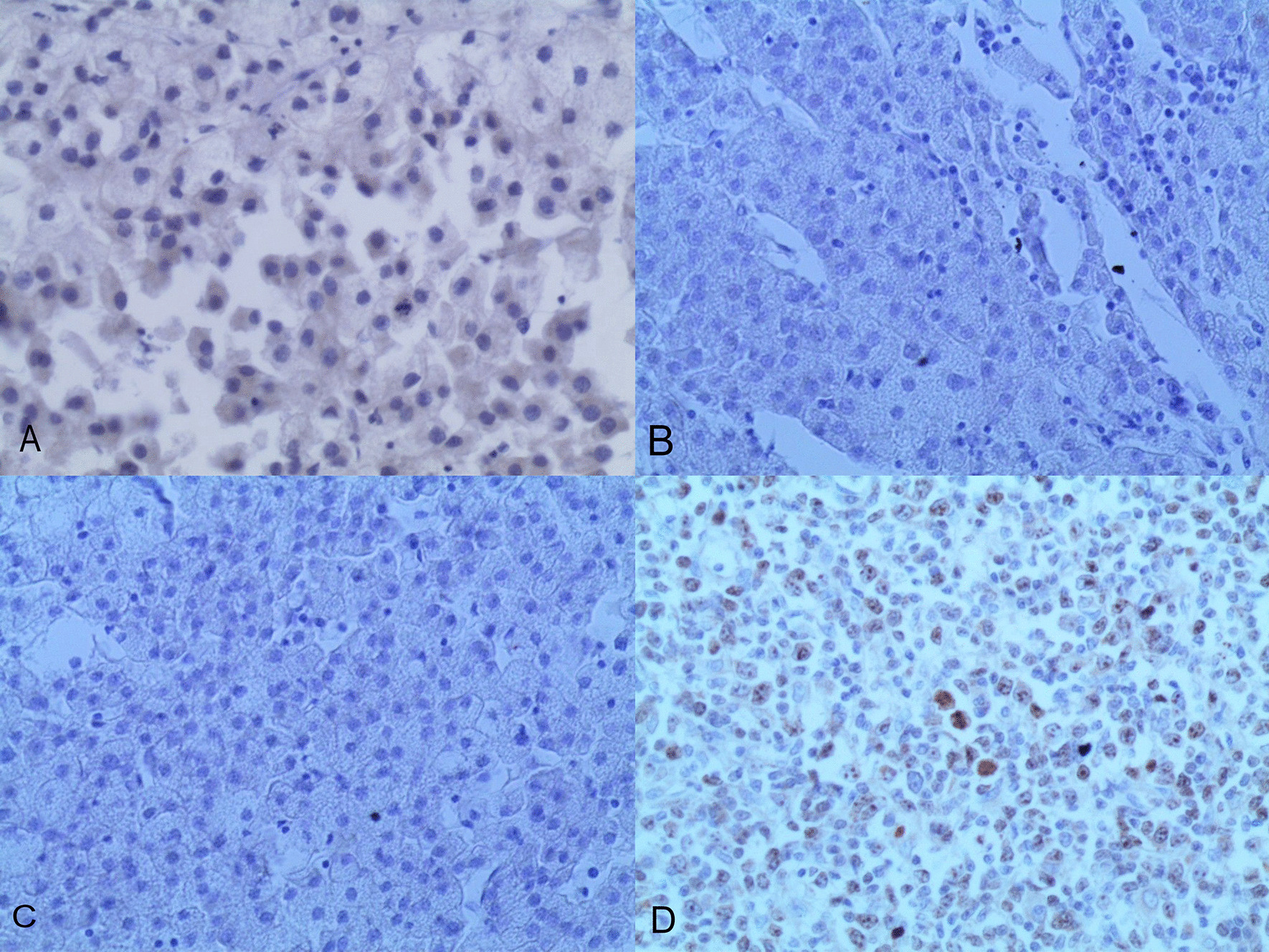
Immunohistochemistry of the neoplasm: **a** Negativity for CD99, **b** negativity for AR, **c** negativity for PR, **d** expression of KI-67: less than 20%)

**Fig. 11:**
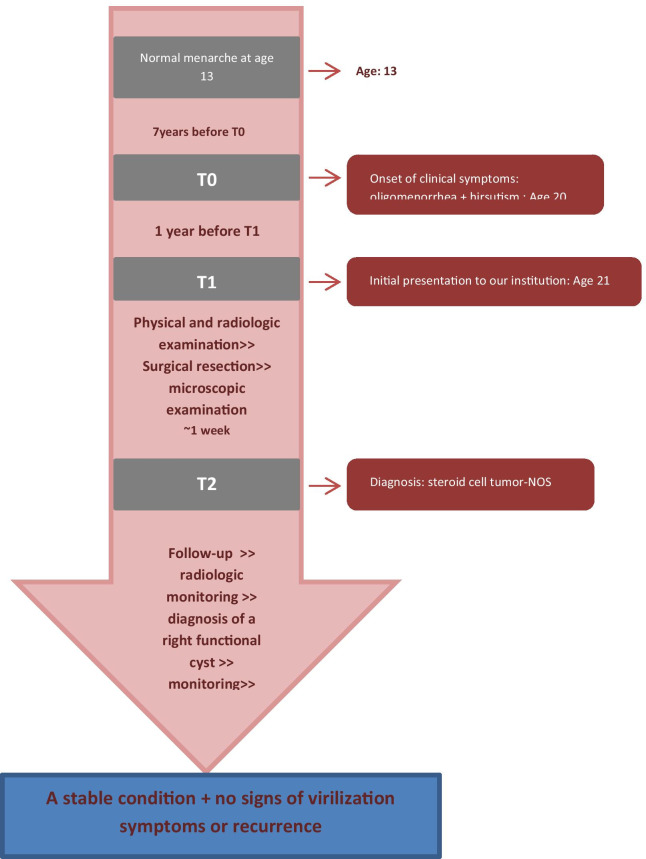
A timeline of the patient’s case

Microscopic examination demonstrated diffuse and nodular proliferation of medium-sized to large polygonal neoplastic cells with pale to granular eosinophilic cytoplasm, small round nuclei, and mild atypia (Fig. 4). The cells were separated by a vascular stroma, with no evidence of capsular invasion (Fig. 5). Few scattered foci of necrosis and hemorrhage were observed (Fig. 6), whereas the mitotic rate was less than 2 per 10 high-power fields, and crystals of Reinke were not observed. Thus final diagnosis was a steroid cell tumor-not otherwise specified (SCT-NOS). Immunohistochemistry revealed positivity for Inhibin-a, Calretinin, and ER (Figs. 7, 8, 9), whereas CD99, PR, and AR showed negative expression, confirming the aforementioned diagnosis, and expression of Ki-67 was estimated to be less than 20% (Fig. 10). Molecular and additional techniques were not available. Following surgery, the patient was hospitalized for 5 days and discharged later with a stable condition and normal laboratory and radiologic results. However, 2 weeks later, she was admitted back to our hospital due to an intermittent localized pain in the right iliac fossa. Full body computed tomography (CT) scan demonstrated a small right ovarian mass that was reported as a benign functional cyst by an expert radiologist and an oncologist, with no other lesions. Due to the patient’s young age and fertility desire, the medical decision was to stay monitored radiographically with no surgical interventions. Two months later, a CT scan demonstrated a prominent decrease in the cyst’s size supporting its benign functional origin. And since her last visit, the patient has been in a stable condition according to clinical and radiologic monitoring with no virilization symptoms. A timeline of the patient’s case can be seen in Fig. 11.

## Discussion

In their largest series of 63 cases of steroid cell tumors-not otherwise specified; Hayes and Scully demonstrated the clinical and morphological characteristics of these neoplasms [1]. SCTs-NOS usually occur in middle-aged women with an average age of 43 years at diagnosis, whereas they are rare in adolescence and younger ages [5]. Our case was described in a 21-year-old female, which represents an additional unique point in our manuscript in addition to being considered the first case report from Syria.

Clinical symptoms usually differ based on hormonal secretion and tumor progression. At early stages, most patients present with virilization signs including amenorrhea or oligomenorrhea as in our case. At later stages, more significant signs of masculinization are presented including severe hirsutism, acne, deepened voice and clitaromegaly [5–7]. Also, estrogenic manifestations have been reported including endometrial hyperplasia and bleeding in postmenopausal women, and in approximately 6–10% of cases, patients might present with symptoms of Cushing’s syndrome due to elevated plasma cortisol levels [1, 7]. Other unspecific symptoms include abdominal pain and distention, whereas patients might be asymptomatic in approximately 25% of cases [7, 8]. In our case, the patient’s complaints were abdominal pain and distention, in addition to the interesting history of hirsutism and oligomenorrhea which—as we suppose—might have been misdiagnosed and treated with oral contraceptives with no further examinations due to the high rate of hormonal dysfunctions in the Mediterranean region [9]. Furthermore, our patient claimed a mild regression in the symptoms, which has increased the difficulties in the diagnosis and management.

Radiologic techniques might be helpful in evaluating gynecologic diseases. According to the literature, ultrasonography, which is typically used in the primary evaluation of ovarian tumors, has limited sensitivity in detecting steroid cell tumors, whereas it could reveal the possibility of malignant transformation through demonstrating abnormal blood flow [7, 10]. Magnetic resonance imaging (MRI) has a higher efficacy in differentiating epithelial and non-epithelial ovarian tumors including steroid cell tumors that appear as intermediate-signal intense heterogeneous solid masses. Also, administration of gadolinium-diethylenetriamine pentaacetic acid (Gd-DTPA) enhancement might increase its sensitivity in detecting primary SCTs as well as metastatic lesions. On the other hand, computed tomography might have a limited role in demonstrating cystic lesions and lipid components [11, 12]. However, in our case, ultrasonographic scanning was performed as a first-line radiological method in the absence of other radiological techniques due to some restrictions. Radiological features in our case suggested a broad spectrum of differential diagnoses. Therefore, while clinical and radiological examinations provide helpful clues for establishing a primary differential diagnosis for ovarian neoplasms, confirming a specific and detailed final diagnosis depends mainly on histopathological examination [13].

Microscopically, steroid cell tumors are characterized by a bimodal proliferation of large polygonal cells with vacuolated cytoplasm and smaller cells with abundant granular eosinophilic cytoplasm. These cells are typically arranged in a diffuse pattern or small nests within the vascular stroma. The absence of crystals or Reinke is helpful to distinguish SCT-NOS from Leidyg cell tumors that are accompanied by Leidyg cell hyperplasia. Also, the lack of spindle cells and fibromatous background is useful to distinguish it from luteinized thecoma. Other differential diagnoses include stromal luteomas that are typically characterized by stromal hyperthecosis involving degenerative pseudovascular spaces, whereas pregnancy luteomas are associated by the proliferation of lutein cells with prominent mitosis in pregnant women, and might present as bilateral or multifocal neoplasms in approximately 33% and 50% of cases respectively. Also, both primary and metastatic clear cell carcinomas could be excluded through the absence of glycogen-rich cytoplasm and eccentric nuclei as in our case [6, 13, 14].

Immunohistochemically, inhibin and calretinin were considered the most useful markers for the differential diagnosis of sex-cord stromal tumors. However, both stains have a low specificity due to the presence of inhibin in ovarian lutein and granulosa cells, as well as the expression of calretinin in neuronal and mesothelial cells. Other immunohistochemical markers include CD99 which is mostly present in neuroectodermal tumors and could be a potential marker for sex-cord stromal tumors due to its presence in granulosa and Sertoli cells [15, 16]. In our case, cells showed positivity for calretinin and inhibin whereas CD99 was negative. Another interesting point in our case was the positive expression of estrogen receptors and negative expression of androgen and progesterone receptors despite the mild virilization symptoms and the normal laboratory findings. These results suggest a possible role for immunohistochemistry with clinicopathological correlation in establishing the final detailed diagnosis.

Staging and prognosis of these neoplasms have been rarely discussed in the literature. Nevertheless, determining malignancy is considered the most essential factor to be assessed as malignant tumors constitute approximately 25–40% of cases. In their largest series of steroid cell tumors, Hayes and Scully have summarized the main histopathological features of malignancy including the presence of more than two mitotic figures per 10 high power fields in 92% of cases, necrosis in 86% of studies, a tumor size larger than 7 cm in 78%, hemorrhage and grade 2–3 atypia in 77% and 64% of cases respectively. Although some cases might present with clinical malignant features including massive ascites, metastases, and satellite nodules, confirming malignancy must be based on the aforementioned pathological features [1, 5, 17, 18]. Interestingly, in our case, we demonstrated a large mass measuring approximately 15 cm in diameter with scattered foci of necrosis and hemorrhage. However, the diagnosis was confirmed as a benign SCT-NOS due to the low mitotic rate of less than 2/10 HPF and the mild atypia, in addition to the benign clinical behavior and the absence of metastatic lesions. Therefore, the decision of malignancy in these neoplasms remains a controversial dilemma, and detailed monitoring and orientation are highly essential.

Treatment decision depends on many prognostic factors including the stage of the tumor, the presence of malignant features, age of the patient, and fertility desire. In general, surgical resection is considered the main choice for benign steroid cell tumors. Therefore, unilateral salpingo-oophorectomy is highly recommended for women with fertility desire and low-stage disease as in our patient, whereas total hysterectomy with bilateral salpingo-oophorectomy is mostly performed in postmenopausal women [5, 6, 19]. Furthermore, adjuvant chemotherapy should be applied to patients with malignant tumors. A study by the Gynecologic Oncology Group has demonstrated a possible role of BEP (Bleomycin, Etoposide, and Cisplatin) in the treatment of malignant ovarian steroid cell tumors [19]. Furthermore, some studies indicated a possible role of GnRH-agonists [20]. Nevertheless, treatment decision remains controversial and more studies are needed to discuss and confirm the best treatment regimen.

## Conclusions

Although ovarian steroid cell tumors represent a rare category, they must be considered in the differential diagnosis for mild virilization symptoms in young females due to the importance of early diagnosis and management. In this manuscript, we aimed to present the first case report of a steroid cell tumor from Syria that highlights the crucial role of detailed morphological examination for challenging cases despite the difficulties in differential diagnosis, and the absence of ancillary techniques.

## Data Availability

All the relevant data and material were presented in the case.

## Abbreviations

SCT-NOS: Steroid cell tumor-not otherwise specified
US: Ultrasonography
CT: Computed tomography
MRI: Magnetic resonance imaging
ER: Estrogen receptor
PR: Progesterone receptor
AR: Androgen receptor
CD: Cluster of differentiation
H&E: Hematoxylin and eosin
